# Supplementary material for: Musculoskeletal Pain in the Neck and Lower Back Regions among PHC Workers: Association between Workload, Mental Disorders, and Strategies to Manage Pain
Source: Healthcare (Basel). 2023 Jan 28;11(3):365. doi: 10.3390/healthcare11030365 (PMC9914445; doi:10.3390/healthcare11030365)
Supplement: Supplementary file 1 [file healthcare-11-00365-s001.zip › Supplementary Materials.pdf]

Musculoskeletal pain in the neck and low back regions among PHC workers: association between workload, mental disorders, and strategies to manage pain (*Article*)

Table S1. Poisson Regression Analysis to assess factors independently associated with neck and low back pain within the previous 12 months

**Outcome**	Factors	**Prevalence ratio (95%CI)**	**P**
Neck pain within the previous 12 months	Marital Status		
	Single	1.00	
	Married/Consensual union	1.20 (0.96 – 1.52)	0.114
	Separated/Divorced	0.92 (0.62 – 1.36)	0.667
	Widowed	1.71 (0.74 – 3.94)	0.208
	Profession		
	Nurse	1.00 (0.73 – 1.36)	0.984
	Physician	1.00	
	Nursing technician/assistant	0.94 (0.64 – 1.39)	0.760
	Community health agent	1.04 (0.79 – 1.37)	0.788
	Dentist	1.67 (1.11 – 2.51)	0.014
	Oral health technician/assistant	1.31 (0.83 – 2.07)	0.246
	Other	2.36 (1.51 – 3.69)	<0.001
	Years of experience in the profession	0.99 (0.96 – 1.01)	0.220
	Years working in a PHC service	1.02 (1.00 – 1.03)	0.038
	Workday in the PHC service		
	Day shift	0.66 (0.39 – 1.15)	0.151
	Night shift	1.00	
	Night/Day shift	0.56 (0.17 – 1.84)	0.335
	Other	1.05 (0.19 – 5.81)	0.959
	Workload		
	Mental Demand	1.02 (0.99 – 1.05)	0.118
	Physical Demand	1.00 (0.98 – 1.02)	0.897
	Temporal Demand	1.00 (0.98 – 1.02)	0.934
	Total effort level	0.99 (0.97 – 1.02)	0.567
	Frustration level	1.03 (1.01 – 1.04)	0.005
	Occupational Risks		
	Physical	1.31 (0.79 – 2.17)	0.290
	Chemical	1.18 (0.92 – 1.52)	0.188
	Ergonomic	1.67 (1.06 – 2.62)	0.026
	Psychosocial	0.94 (0.47 – 1.91)	0.871
	Psychiatric symptoms/disorders	1.88 (1.39 – 2.54)	<0.001
Low back pain within the previous 12 months	Gender		
	Male	1.00	
	Female	1.40 (1.01 – 1.94)	0.042
	Number of children	1.03 (0.97 – 1.09)	0.355
	BMI (kg/m^2)	1.01 (1.00 – 1.02)	0.073
	Years of experience in the profession	1.00 (0.99 – 1.02)	0.767
	Years working in a PHC service	1.00 (0.98 – 1.02)	0.860
	Workload		
	Mental Demand	1.01 (0.99 – 1.03)	0.305
	Total effort level	1.00 (0.98 – 1.02)	0.877
	Frustration level	1.00 (0.99 – 1.02)	0.746
	Occupational Risks		
	Physical	1.21 (0.75 – 1.94)	0.437
	Chemical	1.25 (0.98 – 1.59)	0.070
	Biological	1.38 (0.79 – 2.41)	0.260
	Ergonomic	1.62 (1.06 – 2.48)	0.025
	Psychosocial	1.16 (0.57 – 2.39)	0.683
	Psychiatric symptoms/disorders	1.45 (1.16 – 1.82)	0.001

95%CI= 95% Confidence Interval

Table S2. PHC workers' strategies to manage musculoskeletal pain in the neck and low back regions according to gender.

PHC workers' strategies to manage musculoskeletal pain	Men	Women	P
	n (%)	n (%)	
None	5 (11.1)	23 (7.9)	0.559[a]
Complementary treatment	6 (13.3)	58 (19.9)	0.398[a]
Self-medication	15 (33.3)	110 (37.8)	0.681[a]
Self-care	24 (53.3)	142 (48.8)	0.685[a]
Seeks an Emergency Room	1 (2.2)	4 (1.4)	0.515[b]
Seeks a specialist	9 (20.0)	72 (24.7)	0.614[a]
Seeks a health service	6 (13.3)	85 (29.2)	0.040[a]

[a]Pearson's Chi-Square Test; [b] Fisher's Exact Test.

Table S3. PHC workers' strategies to manage musculoskeletal pain in the neck and low back regions according to educational level.

PHC workers' strategies to manage musculoskeletal pain	Complete High School	Some undergraduate studies/bachelor's degree/ Technician	Specialization/ Master's/ Ph.D.	P[a]
	n (%)	n (%)	n (%)	
**None**	12 (9.3)	11 (8.1)	5 (6.9)	0.839
Complementary care	18 (14.0)	26 (19.1)	21 (29.2)*	0.032
Self-medication	46 (35.7)	48 (35.3)	32 (44.4)	0.377
Self-care	59 (45.7)	68 (50.0)	39 (54.2)	0.506
Seeks Emergency Rooms	4 (3.1)	1 (0.7)	0 (0.0)	0.141
Seeks a specialist	36 (27.9)	25 (18.4)	21 (29.2)	0.109
Seeks healthcare units	47 (36.4)*	33 (24.3)	11 (15.3)	0.003

* Statistically significant association according to residuals adjusted at 5% of significance; [a] Pearson's Chi-Square.

Table S4. PHC workers' strategies to manage musculoskeletal pain in the neck and low back regions according to the profession.

PHC workers' strategies to manage musculoskeletal pain	Nurse	Physician	Nursing technician/assistant	Health Community Agent	Dentist	Oral health technician/assistant	p[a]
	n (%)	n (%)	n (%)	n (%)	n (%)	n (%)	
**None**	5 (10.0)	3 (7.0)	3 (4.2)	15 (10.9)	1 (7.7)	1 (6.7)	0.717
Complementary care	17 (34.0)*	7 (16.3)	11 (15.3)	23 (16.7)	3 (23.1)	0 (0.0)	0.002
Self-medication	21 (42.0)	25 (58.1)*	24 (33.3)	41 (29.7)	5 (38.5)	8 (53.3)	0.029
Self-care	27 (54.0)	24 (55.8)	37 (51.4)	60 (43.5)	8 (61.5)	6 (40.0)	0.520
Seeks Emergency Rooms	0 (0.0)	0 (0.0)	2 (2.8)	3 (2.2)	0 (0.0)	0 (0.0)	0.785
Seeks a specialist	11 (22.0)	4 (9.3)	25 (34.7)*	27 (19.6)	5 (38.5)*	6 (40.0)*	0.002
Seeks healthcare units	7 (14.0)	1 (2.3)	19 (26.4)	55 (39.9)*	2 (15.4)	7 (46.7)*	<0.001

* Statistically significant association according to residuals adjusted at 5% of significance; [a] Pearson's Chi-Square.

Table S5. PHC workers' strategies to manage musculoskeletal pain in the neck and low back regions according to the presence of mental disorders.

PHC workers' strategies to manage musculoskeletal pain	Mental Disorders		P
	Yes	No	
	n (%)	n (%)	
None	15 (6.2)	13 (13.5)	0.048[a]
Complementary care	51 (21.2)	14 (14.6)	0.219[a]
Self-medication	109 (45.2)	17 (17.7)	<0.001[a]
Self-care	123 (51.0)	43 (44.8)	0.361[a]
Seeks Emergency Rooms	5 (2.1)	0 (0.0)	0.327[b]
Seeks a specialist	62 (25.7)	20 (20.8)	0.421[a]
Seeks healthcare units	68 (28.2)	23 (24.0)	0.510[a]

[a]Pearson's Chi-Square; [b] Fisher's Exact Test.

Table S6. Health workers' strategies to manage musculoskeletal pain in the neck and low back regions according to time working in PHC services.

PHC workers' strategies to manage musculoskeletal pain	Time working in PHC services*		P
	<8 years	≥ 8 years	
	n (%)	n (%)	
None	14 (9.0)	14 (7.8)	0.829[a]
Complementary care	19 (12.3)	45 (25.0)	0.005[a]
Self-medication	54 (34.8)	71 (39.4)	0.450[a]
Self-care	81 (52.3)	84 (46.7)	0.362[a]
Seeks Emergency Rooms	0 (0.0)	5 (2.8)	0.064[b]
Seeks a specialist	29 (18.7)	52 (28.9)	0.041[a]
Seeks healthcare units	33 (21.3)	56 (31.1)	0.057[a]

* Cut off point determined by the median; [a] Pearson's Chi-Square; [b] Fisher's Exact Test.
